# Seasonal variation in glucose and insulin is modulated by food and temperature conditions in a hibernating primate

**DOI:** 10.3389/fphys.2023.1251042

**Published:** 2023-09-07

**Authors:** Marina B. Blanco, Lydia K. Greene, Laura N. Ellsaesser, Cathy V. Williams, Catherine A. Ostrowski, Megan M. Davison, Kay Welser, Peter H. Klopfer

**Affiliations:** ^1^ Duke Lemur Center, Durham, NC, United States; ^2^ Department of Biology, Duke University, Durham, NC, United States

**Keywords:** *Cheirogaleus*, dwarf lemur, hibernation, thermoconforming, torpor

## Abstract

Feast-fast cycles allow animals to live in seasonal environments by promoting fat storage when food is plentiful and lipolysis when food is scarce. Fat-storing hibernators have mastered this cycle over a circannual schedule, by undergoing extreme fattening to stockpile fuel for the ensuing hibernation season. Insulin is intrinsic to carbohydrate and lipid metabolism and is central to regulating feast-fast cycles in mammalian hibernators. Here, we examine glucose and insulin dynamics across the feast-fast cycle in fat-tailed dwarf lemurs, the only obligate hibernator among primates. Unlike cold-adapted hibernators, dwarf lemurs inhabit tropical forests in Madagascar and hibernate under various temperature conditions. Using the captive colony at the Duke Lemur Center, we determined fasting glucose and insulin, and glucose tolerance, in dwarf lemurs across seasons. During the lean season, we maintained dwarf lemurs under stable warm, stable cold, or fluctuating ambient temperatures that variably included food provisioning or deprivation. Overall, we find that dwarf lemurs can show signatures of reversible, lean-season insulin resistance. During the fattening season prior to hibernation, dwarf lemurs had low glucose, insulin, and HOMA-IR despite consuming high-sugar diets. In the active season after hibernation, glucose, insulin, HOMA-IR, and glucose tolerance all increased, highlighting the metabolic processes at play during periods of weight gain *versus* weight loss. During the lean season, glucose remained low, but insulin and HOMA-IR increased, particularly in animals kept under warm conditions with daily food. Moreover, these lemurs had the greatest glucose intolerance in our study and had average HOMA-IR values consistent with insulin resistance (5.49), while those without food under cold (1.95) or fluctuating (1.17) temperatures did not. Remarkably low insulin in dwarf lemurs under fluctuating temperatures raises new questions about lipid metabolism when animals can passively warm and cool rather than undergo sporadic arousals. Our results underscore that seasonal changes in insulin and glucose tolerance are likely hallmarks of hibernating mammals. Because dwarf lemurs can hibernate under a range of conditions in captivity, they are an emerging model for primate metabolic flexibility with implications for human health.

## 1 Introduction

Hibernation is a metabolic strategy used to cope with energetic crises like seasonal food scarcity (Ruf and Geiser, 2015). Among mammals, hibernation has evolved independently in members of all major lineages (Carey et al., 2003) with thermolability proposed as the ancestral condition (Lovegrove, 2012a; Lovegrove, 2012b). Depending on the species and local environment there is considerable variation in hibernation “styles” (Canale and Henry, 2010; Geiser and Körtner, 2010; Canale et al., 2012; Staples, 2016). For example, small-bodied, temperate hibernators tend to cycle between multi-day torpor bouts at low temperature and brief arousals to euthermia (Carey et al., 2003; Geiser, 2013); large bears tend to depress metabolism for prolonged periods while maintaining warm body temperature (Tøien et al., 2011); and tropical hibernators can depress metabolism under a range of ambient temperature conditions, from stable cold to those that fluctuate considerably across the day (Canale et al., 2012; Dausmann et al., 2012; van Breukelen and Martin, 2015). Despite this variation in metabolism across species, one commonality shared among fat-storing hibernators is the seasonal cycle between fat deposition and fat depletion (Carey et al., 2003; Giroud et al., 2021). This consistency between feast and fast cycles raises questions about the mammalian mechanisms that regulate switches between carbohydrate and lipid metabolism (Andrews, 2007; Giroud et al., 2021).

At the center of energy balance in mammals is insulin, a peptide hormone that maintains glucose homeostasis and is involved in lipid and protein metabolism (Wilcox, 2005; Keane and Newsholme, 2014). In the broadest terms, glucose enters the bloodstream, which triggers beta cells in the pancreas to secrete insulin, which in turn, promotes the uptake of surplus glucose by muscle or adipose tissues for storage as glycogen or lipids (Keane and Newsholme, 2014). Insulin also inhibits lipolysis (Sears and Perry, 2015). To burn stored lipids, target tissues can become insulin insensitive or insulin resistant (Sears and Perry, 2015). Thus insulin, directly or indirectly, affects how both carbohydrate- and lipid-based energy is synthesized, stored, and metabolized over a lifetime (Wilcox, 2005). In addition to insulin, other hormones and signaling molecules play essential roles in metabolic regulation (e.g., glucagon, ghrelin, leptin, *etc.*) (Healy et al., 2010; Amitani et al., 2013; Weitten et al., 2013). For hibernators, however, there is considerable literature on glucose, insulin, and reversible insulin resistance, in large part, to probe how hibernators can naturally withstand seasonal obesity and starvation, conditions that can be detrimental in non-hibernators, like humans (Martin, 2008; Wu et al., 2013).

In certain hibernating rodents, the fattening season prior to hibernation is characterized by greater concentrations of circulating glucose and insulin (Florant and Greenwood, 1986; Tokuyama et al., 1991; Martin, 2008), as well as insulin insensitivity (Melnyk et al., 1983; Florant et al., 1985; Florant and Greenwood, 1986; Tokuyama et al., 1991). In these systems, insulin sensitivity is then restored after hibernation has ended (Martin, 2008; Wu et al., 2013). Hedgehogs and bears, by contrast, show reduced circulating glucose and/or insulin during the fattening season, as well as increased insulin sensitivity (Laurila and Suomalainen, 1974; Rigano et al., 2017). Like rodents, bears are also sensitive to insulin after hibernation (Rigano et al., 2017). These differences between species during the fattening season could perhaps be related to differences in seasonal diets, the degree of fat deposition at the time of sampling, or experimental study design.

During hibernation, hibernators generally show more consistent patterns of glucose, insulin, and insulin sensitivity. For animals cycling between torpor and arousal, circulating glucose and insulin levels increase as animals rewarm and ratchet-up metabolism (Konttinen et al., 1964; Tashima et al., 1970; Galster and Morrison, 1975; Hoo-Paris et al., 1978; Castex and Sutter, 1979; Hoo-Paris and Sutter, 1980; Al-Badry and Taha, 1983). During hibernation, a time when significant lipolysis is essential to fuel metabolism, insulin insensitivity is common to rodents (Moreau-Hamsany et al., 1988), hedgehogs (Hoo-Paris et al., 1978), and bears (McCain et al., 2013; Rigano et al., 2017). The idea that greater insulin insensitivity in mammalian heterotherms is necessary for lipolysis is further supported from studies using rodents that normally hibernate, but that were artificially kept under warm conditions, constant photoperiod, and with food year-round: During periods of weight loss (i.e., fat burn), the animals did not differ in circulating glucose or insulin relative to periods of weight gain, but they did show greater insulin insensitivity (Grimes et al., 1981; Melnyk et al., 1983; Melnyk and Martin, 1985). Likewise, mouse lemurs that are facultative hibernators from Madagascar and gain weight seasonally in captivity without expressing hibernation, showed greater insulin sensitivity and insensitivity respectively during periods of weight gain and loss (Terrien et al., 2018; Giroud et al., 2021). These primates also had the greatest concentrations of circulating insulin when losing weight, with no correlational change in glucose (Terrien et al., 2018), perhaps due to the continued availability of food.

The fat-tailed dwarf lemur (*Cheirogaleus medius;* 150–300 g) is a larger cousin of the mouse lemur and presents a fascinating system for probing heterothermic flexibility and metabolic correlates in an obligate hibernator. Fat-tailed dwarf lemurs are endemic to the dry deciduous forests of western Madagascar, where they hibernate during the dry season (Fietz and Dausmann, 2006). In anticipation of hibernation, these lemurs almost double their body mass during the ∼2-month fattening period by eating ripe fruits and converting the fruit sugar into fat that is deposited primarily around their tail (Fietz and Ganzhorn, 1999). As tropical hibernators, fat-tailed dwarf lemurs can hibernate under a range of conditions, from stable temperatures of 12°C–20°C to those that fluctuate daily from 10°C–30°C. Under stable temperatures, fat-tailed dwarf lemurs cycle between multi-day torpor bouts (in which body temperature approximates ambient) and periodic arousals. Under fluctuating conditions, they thermoconform, i.e., passively track warming and cooling ambient temperatures without fully arousing, for weeks at a time (Dausmann et al., 2004; Dausmann et al., 2005).

The Duke Lemur Center (DLC) in Durham, NC houses the only reproductive population of fat-tailed dwarf lemurs outside of Madagascar. Unlike mouse lemurs, fat-tailed dwarf lemurs can hibernate under appropriate laboratory conditions (Blanco et al., 2021; Blanco et al., 2022a). During the lean season, DLC dwarf lemurs can be maintained in warm rooms with available food, which maximally allows only shallow metabolic depression, or in temperature-controlled “hibernacula” rooms where food can be offered or withheld, depending on the experimental setup. The hibernacula can be set to different temperature profiles, including stable or fluctuating conditions. Taken together, this setup allows for experimental studies to track metabolic signatures across seasons and within the lean season relative to environmental condition.

In the present study, we add to the literature on glucose and insulin dynamics in hibernators using the natural metabolic flexibility of the fat-tailed dwarf lemur and the experimental setup at the DLC. Specifically, we combine data on circulating glucose and insulin assayed from the DLC dwarf lemur colony during the fattening, lean, and active seasons of two years. During the lean season, we measured these analytes in animals housed under various temperature and food regimens, and thus expressing a range of metabolic strategies. Lastly, we conducted glucose tolerance tests on a subset of subjects across seasons. Under the hypothesis that glucose intolerance and insulin insensitivity are hallmarks of lipid-based metabolism and seasonally required for fat-storing hibernators, we predict that dwarf lemurs will show signatures consistent with lean-season insulin insensitivity, with variation across metabolic strategies and states.

## 2 Materials and methods

### 2.1 Subjects and housing

The subjects were 28 fat-tailed dwarf lemurs that ranged in age from 0.3–16 years and were studied in 2021–2022 and 2022–2023. In year 1, the subjects included 23 lemurs (10 adult males; 5 adult females; 5 juvenile males; 3 juvenile females); in year 2, the subjects included 20 lemurs (11 adult males; 4 adult females; 1 juvenile male; and 4 juvenile females). A subset of 15 subjects participated in both years. We define juveniles as animals under 3 years of age (Blanco and Godfrey, 2013).

DLC dwarf lemurs are housed in small family units or solitarily in indoor enclosures year-round. Water is always freely available. They are all maintained under an artificial, North Carolina-like photoperiod (Blanco et al., 2021) with the onset of the light cycle occurring at 11:30a.m. To accommodate for a changing photoperiod, the light phase is shortened or lengthened by ∼15–30 min every other week, with the shortest light:dark cycle being 9.5 h:14.5 h (starting in late fall) and longest 14.5 h:9.5 h (starting in late spring).

We define mid-September to mid-November as the core of the fattening season, December-February as the core of the lean season, and March-May as the active season.

During the fattening and active seasons, dwarf lemurs are maintained under stable warm conditions (22°C–25°C). During the fattening season, they are fed a high-sugar diet (∼15 g fresh fruit, ∼3 g monkey biscuit, 2 g dried fruit), whereas during the active season they are fed a high-fat diet (∼12 g fruit and veggie mix, 6 g monkey biscuit, 2 mealworms) (Blanco et al., 2022b). This regimen mimics the natural foraging patterns of wild populations (Fietz and Ganzhorn, 1999) and helps mediate seasonal fat deposition and depletion (Blanco et al., 2022b).

**FIGURE 1:**
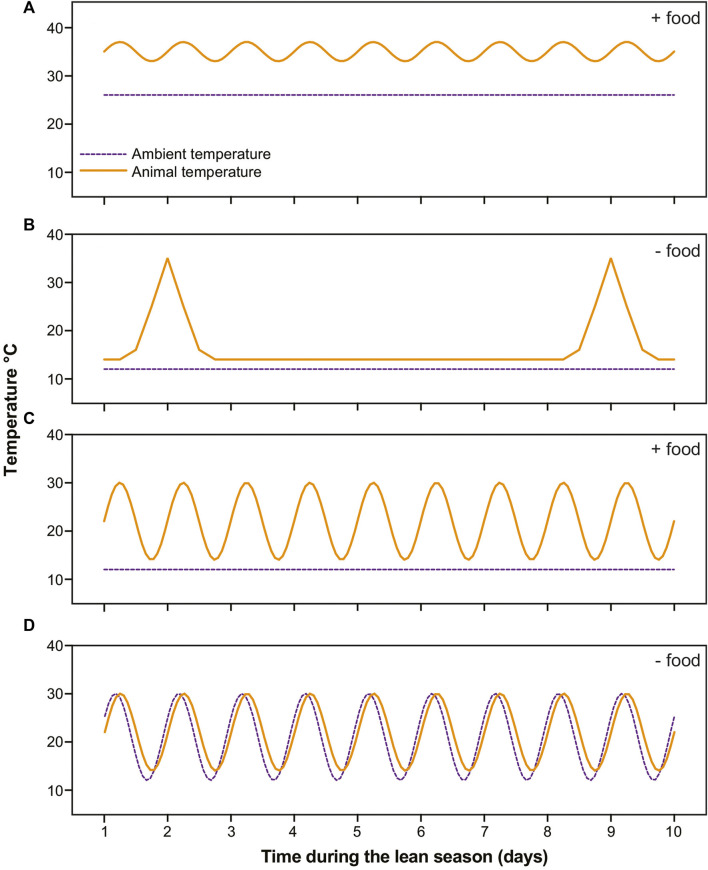
Schematics of temperature profiles in dwarf lemurs (solid orange lines) and rooms (dashed purple lines) in the four categories during 10 days in the lean season. **(A)** Lemurs under stable warm conditions with food undergo shallow torpor; **(B)** Lemurs under stable cold conditions without food cycle between multiday bouts of torpor and brief arousals; **(C)** Lemurs under stable cold conditions with food availability undergo daily torpor; **(D)** Lemurs under fluctuating daily conditions thermoconform, that is, passively track ambient temperatures without consistently using thermogenesis to arouse.

During the lean season, six of our subjects remained under stable warm conditions and were fed a calorie-reduced, high-fat diet (Blanco et al., 2022b). The remaining subjects were transferred to hibernacula rooms. Of these, 16 were housed under stable cold conditions (∼15°C) and underwent food deprivation: These animals cycled between multiday torpor bouts and periodic arousals, i.e., they hibernated (Blanco et al., 2022a). An additional 9 subjects were housed under stable cold conditions but offered food daily: These subjects routinely ate and showed daily torpor expression (Blanco et al., unpublished data). The remaining 10 subjects were housed under fluctuating temperature conditions that cycled from 12°C–30°C daily. These subjects were food deprived and consistently thermoconformed (Blanco et al., unpublished data) (Figure 1).

Dwarf lemurs are endangered primates: In line with the DLC’s mission, we designed our study to be minimally invasive and non-harmful to the animals, which understandably limited our experimental options. This study was approved by the DLC’s Research Committee and Duke University’s Institutional Animal Care and Use Committee (protocol A213-20-11).

### 2.2 Glucose tolerance tests

We conducted glucose tolerance tests in year 1 on a subset of animals during the fattening (4 adults; 4 juveniles), lean (3 adults; 1 juvenile), and active (6 adults; 5 juveniles) seasons, as well as control tests in the fattening (4 adults), lean (2 adults), and active (5 adults; 1 juvenile) seasons. Animals were assigned to either test or control conditions and always underwent the same experimental procedure when studied at different timepoints. Because of concerns to animal welfare, we only performed lean-season tolerance tests on animals maintained under warm conditions with food.

Glucose and control tests were conducted in the morning hours in the DLC’s veterinary clinic during the final hours of the light phase (∼8:00–11:30a.m.) following an overnight fast. Individuals were manually restrained and anesthetized. We chose Telazol (10.5 mg/kg, IM) because it has minimal interference with glucose metabolism (Gresl et al., 2000; Vaughan et al., 2014). Once the lemurs were anesthetized, we collected a fasting blood sample in EDTA from the tail or saphenous vein for insulin, from which we aliquoted two drops of whole blood to measure blood glucose in duplicate using a handheld glucometer (Contour next EZ, Ascensia, Inc.). The remaining whole blood was promptly spun to plasma and stored at −80°C until analysis.

We waited 15 min after anesthesia administration to begin tests. For the glucose tests, we administered 5% dextrose in sterile water (40 mL/kg, subcutaneous injection); for the control tests, we used sterile saline solution (0.9% NaCl, subcutaneous injection), with the volume adjusted per individual weight. We collected serial blood drops at the 15-, 30-, 60-, and 90-min marks to measure glucose in duplicate using the glucometer. Once the tests were completed, animals recovered in transport kennels and were returned to their home enclosure during the first part of their active phase.

### 2.3 Biological sampling

We collected blood samples for glucose and/or insulin from study lemurs not undergoing tolerance tests in year 1 and all animals in year 2. During the fattening and active seasons, and for animals kept under warm conditions during the lean season, dwarf lemurs were sampled during the final hours of their light phase following an overnight fast. They were manually restrained and anesthetized (Ketamine; 10 mg/kg IM). We collected a blood sample in EDTA, aliquoted drops to measure glucose, and stored the remainder as plasma, as described above.

For lean-season dwarf lemurs in the hibernacula rooms of year 1, we measured glucose from animals under stable cold conditions with and without food while animals were torpid and aroused. In the early morning (7:00a.m.), we confirmed if study animals were torpid by checking temperature recordings from lemurs’ external transmitters. Dwarf lemurs in the hibernacula rooms wore temperature-sensitive radiocollars that allowed us to determine individuals’ metabolic status (for more details on radiocollars, see Blanco et al., 2021). If torpid, we restrained dwarf lemurs close to their home enclosure without anesthesia for rapid sampling of blood drops to measure glucose. These animals were placed in transport kennels in warm rooms for several hours. Once aroused, they were anesthetized for an additional blood draw to measure blood glucose. For lean-season dwarf lemurs in the hibernacula rooms of year 2, we brought lemurs to the clinic in transport kennels in the early morning (7:00a.m.), where they were housed for several hours. Once aroused, they underwent full blood sampling under anesthesia for glucose and insulin as described above.

All animals were allowed to recover from anesthesia in transport kennels and warm rooms. Animals were returned to their home enclosures during the first part of their active phase.

### 2.4 Sample and statistical analyses

For glucose, we averaged values across duplicate droplets. In total, we measured blood glucose in 36 samples from animals during the fattening season, in 53 samples from animals during the lean season, and in 40 samples from animals during the active season (Table 1). For collected plasma, we submitted 50 μ L aliquots for insulin assays to Eurofins SF Analytical DBA Craft Technologies (ELISA NBP2-60076-1 kit). In total, we measured insulin in 32 samples from animals during the fattening season, in 24 samples from aroused/active animals during the lean season, and in 32 samples from animals during the active season (Table 1). All insulin values had a paired glucose value: We calculated the homeostatic model assessment for insulin resistance (HOMA-IR) following the formula = fasting insulin [µIU/mL] × fasting glucose [mg/dL])/405 (Terrien et al., 2018).

**TABLE 1:** The number of samples across seasons, conditions, and metabolic states analyzed for glucose and insulin.

Assay type	Fattening	Lean						Active
		Cold; no food		Cold; food		Fluctuating; no food	Warm; food	
		Torpid	Aroused	Torpid	Aroused			
Glucose	36	8	16	5	8	10	6	40
Insulin	32	NA	8	NA	1	10	5	32
HOMA-IR	32	NA	8	NA	1	10	5	32

We implemented a series of linear mixed models (LMMs) via the lmerTest (version 3.1–3; Kuznetsova et al., 2017) package in Rstudio (version 2022.07.2; RStudio Team, 2022) with R software (version 4.2.1; R Core Team, 2022). We used log-transformed data, as they improved model fit as assessed by reduced AIC scores. For glucose, insulin, and HOMA-IR, we first computed models within the fattening season to determine whether animals placed under different conditions during the ensuing lean season showed any differences. We used glucose, insulin, or HOMA-IR as the dependent variable, lean-season condition, study year, animal sex and age (in years) as the independent variables, and individual lemur as a random term.

Next, we compared differences across seasons, regardless of what condition animals were placed under during the lean season. We excluded glucose values from torpid animals. We included glucose, insulin or HOMA-IR as the dependent variable, season, animal sex and age as the independent variables, and lemur as a random term. Within the lean season, we compared differences across conditions. We computed LMMs by entering glucose, insulin, or HOMA-IR as the dependent variable, lean-season condition as the independent variable, and lemur as a random term. We excluded sex and age as explanatory variables for models within seasons, given the reduced sample sizes. We excluded glucose values from torpid animals, and the single insulin value from the individual under cold conditions with food.

We asked if glucose differed between torpid and aroused animals. We ran a LMM for the lemurs kept under cold conditions with and without food, using glucose as the dependent variable, metabolic state (torpid *versus* aroused) nested within lean-season condition (cold, with *versus* without food) as the independent variable, and lemur as the random term.

For the glucose and control tolerance tests, we computed the area under the curve (AUC) across sampling timepoints per test in GraphPad Prism (version 9.5.0). We used log (AUC) values as they improved model fit. We compared experimental vs control tests using a LMM in which AUCs were entered as the dependent variable, treatment status as the independent variable, and lemur as the random term. Next, we looked within experimental subjects to ask if AUCs differed across seasons. We computed a LMM with AUC entered as the dependent variable, season as the independent variable, and lemur as the random term. Lastly, we asked if there was an effect of age. Due to sampling sizes, we restricted this model to only the fattening and active seasons: We included AUCs as the dependent variable, age category (adult vs juvenile) nested within season as the independent variable, and lemur as a random term.

## 3 Results

### 3.1 Results in the fattening season

Within the fattening season, neither fasting blood glucose (t = 0.168, *p* = 0.868), insulin (t = 1.228, *p* = 0.254), nor HOMA-IR (t = 0.955, *p* = 0.364) varied by study year. Likewise, forthcoming lean-season condition was not significantly associated to glucose (t < 1.555, *p* > 0.131 for all pairwise comparisons) or HOMA-IR (t < 1.714, *p* > 0.112 for all pairwise comparisons). Insulin showed no difference during the fattening season in lemurs that would experience cold conditions without vs with food (t = 1.604, *p* = 0.145) or vs fluctuating conditions without food (t = 1.475, *p* = 0.154). We did find a difference during the fattening season between lemurs that would experience cold conditions with food and fluctuating conditions without food (t = 2.384, *p* = 0.039).

During the fattening season, males had significantly greater glucose than did females (t = 2.919, *p* = 0.007), and older animals trended towards having decreasing glucose (t = −1.833, *p* = 0.077). We found no such associations between insulin and animal sex (t = 1.216, *p* = 0.243) and age (t = 0.633, *p* = 0.534) or between HOMA-IR and animal sex (t = 0.537, *p* = 0.601) and age (t = 0.129, *p* = 0.899).

### 3.2 Results across seasonal timepoints

**FIGURE 2:**
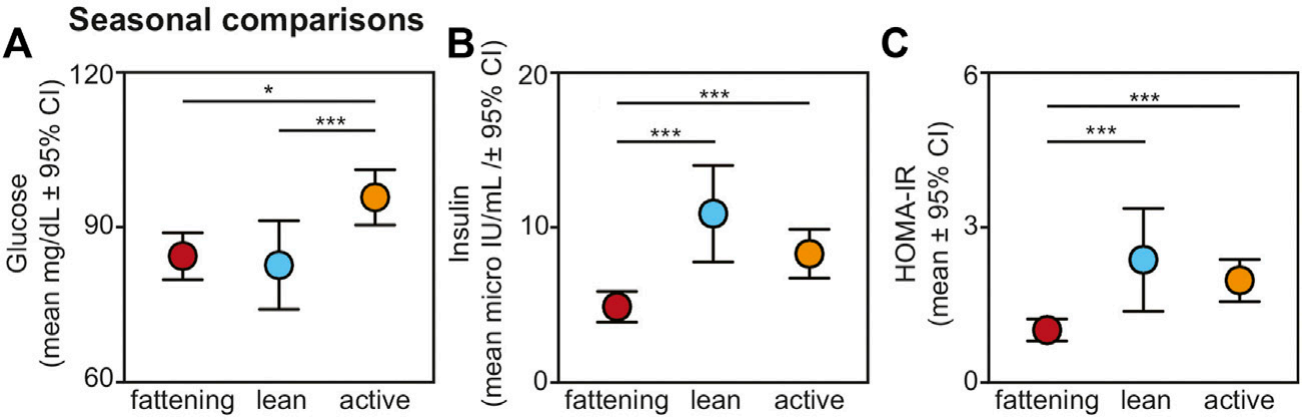
Fasting **(A)** glucose, **(B)** insulin, and **(C)** HOMA-IR values from non-torpid dwarf lemurs sampled across the fattening (red), lean (blue), and active (orange) seasons. All metrics are graphed as mean units ± 95% confidence intervals. *p < 0.05; ***p < 0.001.

We found an overall, strong effect of season on glucose, insulin, and HOMA-IR values (Figure 2). Regarding glucose, lemurs during the active season had greater values compared to the fattening (t = 2.474, *p* = 0.015) and lean (t = 3.660, *p* < 0.001) seasons (Figure 2A). There was no difference in blood glucose between the fattening and lean seasons (t = 1.088; *p* = 0.279).

Insulin varied across seasons, with significantly reduced values in the fattening season compared to the lean (t = −4.484, *p* < 0.001) and active (t = −3.767, *p* < 0.001) seasons (Figure 2B). There was no difference in insulin between the lean and active seasons (t = 1.005, *p* = 0.318). HOMA-IR varied seasonally similarly to insulin: HOMA-IR was lower in the fattening season compared to the lean (t = 3.483, *p* < 0.001) and active (t = 4.120, *p* < 0.001) seasons (Figure 2C), and there was no difference between the lean and active seasons (t = 0.323, *p* = 0.747). Average HOMA-IR values were 0.99 for the fattening season, 2.36 for the lean season, and 1.96 for the active season.

For glucose, sex remained a significant effect in this seasonal model, with males having greater glucose values compared to females (t = 2.236; *p* = 0.027), but when considering this fuller sample set, we found no effect of animal age (t = −0.818, *p* = 0.415). Neither sex nor age were significantly associated with either insulin or HOMA-IR in these fuller models (t < 0.985, *p* > 0.336 for all comparisons).

### 3.3 Results across lean-season conditions

**FIGURE 3:**
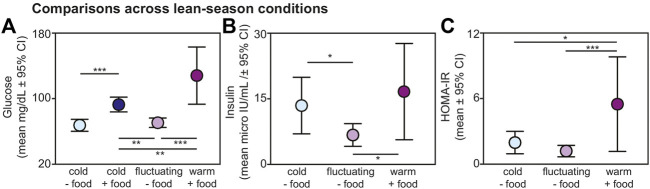
Fasting **(A)** glucose, **(B)** insulin, and **(C)** HOMA-IR values from non-torpid dwarf lemurs under different conditions during the lean season, including cold conditions without food (light blue), cold conditions with food (dark blue), fluctuating conditions without food (light purple), and warm conditions with food (dark purple). All metrics are graphed as mean units ± 95% confidence intervals. *p < 0.05*;* **p < 0.01; ***p < 0.001.

Within the lean season, we found effects of ambient temperature and food availability on glucose, insulin, and HOMA-IR values from non-torpid animals (Figure 3). Regarding glucose, lemurs without provisioned food did not differ in blood glucose when housed under cold *versus* fluctuating temperatures (t = 0.721; *p* = 0.476); however, lemurs under cold conditions without food had significantly reduced glucose compared to lemurs housed under cold conditions with food (t = −4.271; *p* < 0.001) and compared to those housed under warm conditions with food (t = −7.211; *p* < 0.001) (Figure 3A). Likewise, lemurs under fluctuating conditions without food had reduced glucose values compared to lemurs under cold conditions with food (t = −3.356, *p* = 0.005) and compared to those under warm conditions with food (t = −6.324, *p* < 0.001). Lemurs housed under cold conditions with food had reduced glucose compared those under warm conditions with food (t = −3.026, *p* = 0.005).

From non-torpid animals within the lean season, insulin was significantly reduced in lemurs maintained under fluctuating conditions without food compared to those under cold conditions without food (t = −2.480, *p* = 0.022) and under warm conditions with food (t = −2.718, *p =* 0.013) (Figure 3B). There was no difference in insulin between animals under cold conditions without food and warm conditions with food (t = 0.548, *p* = 0.590) and both groups showed considerable variation in fasting insulin.

Regarding HOMA-IR, values were significantly greater in animals maintained under warm conditions with food compared to those under cold conditions without food (t = 2.482, *p* = 0.022) and fluctuating conditions without food (t = 3.974, *p* < 0.001) (Figure 3C). We found no difference in HOMA-IR values between lemurs under cold and fluctuating conditions without food (t = 1.606, *p* = 0.124). Importantly, average HOMA-IR values were 1.95 for non-torpid lemurs under cold conditions without food, 1.17 for non-torpid lemurs under fluctuating conditions without food, and 5.49 for non-torpid lemurs under warm conditions with daily food. Thus, HOMA-IR values in dwarf lemurs housed in warm rooms with food are well above the standard threshold of 2.0 for insulin resistance.

**FIGURE 4:**
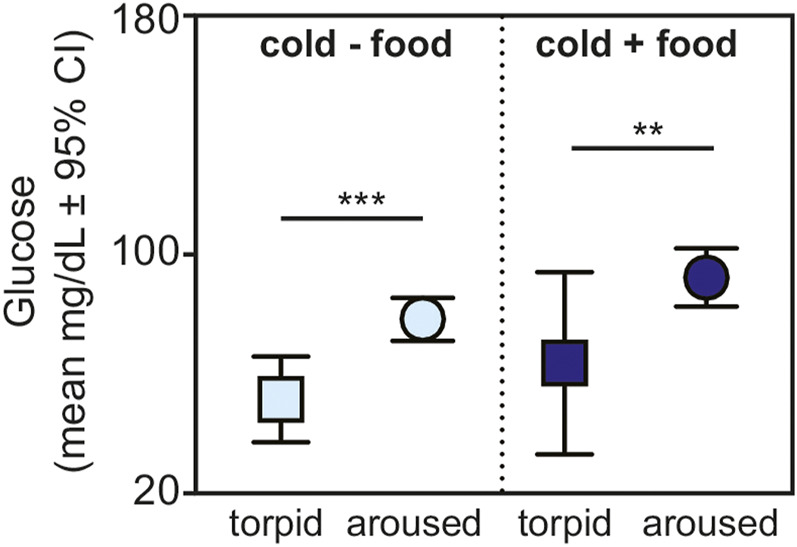
Fasting glucose in torpid (square) and aroused (circle) dwarf lemurs during the lean season maintained under cold conditions without food (light blue) and with food (dark blue). All metrics are graphed as mean units ± 95% confidence intervals. **p < 0.01; ***p < 0.001.

Lastly, for animals housed under cold conditions, we found greater glucose values in aroused *versus* torpid animals for those without (t = 3.956, *p* < 0.001) and with (t = 3.080, *p* = 0.005) available food (Figure 4).

### 3.4 Results of glucose tolerance tests

**FIGURE 5:**
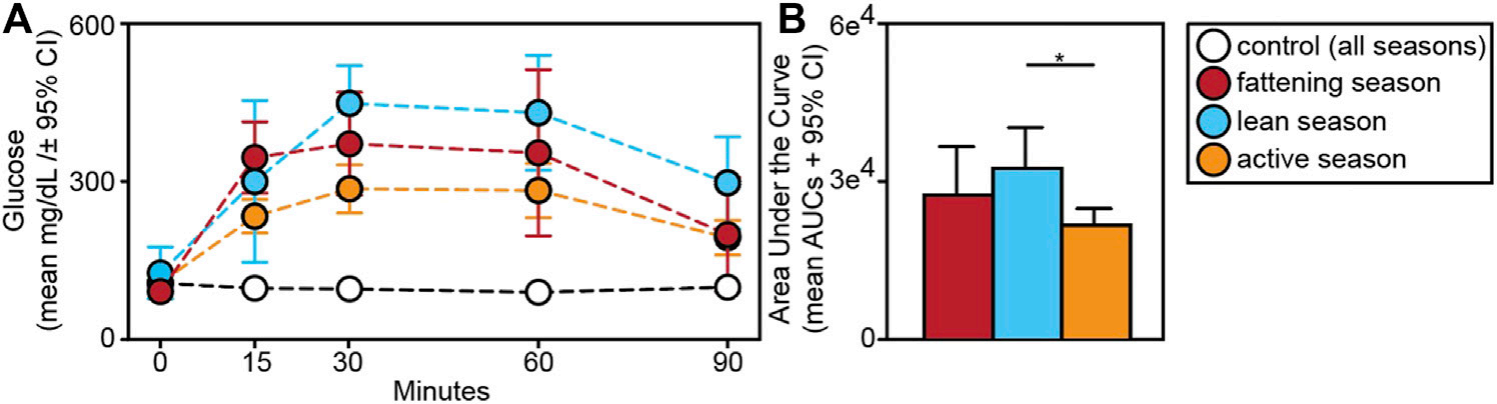
**(A)** Glucose tolerance test profiles from dwarf lemurs given a dextrose challenge during the fattening, lean and active season or given a control saline solution; **(B)** Area under the curve for dwarf lemurs sampled across seasons. *p < 0.05.

**FIGURE 6:**
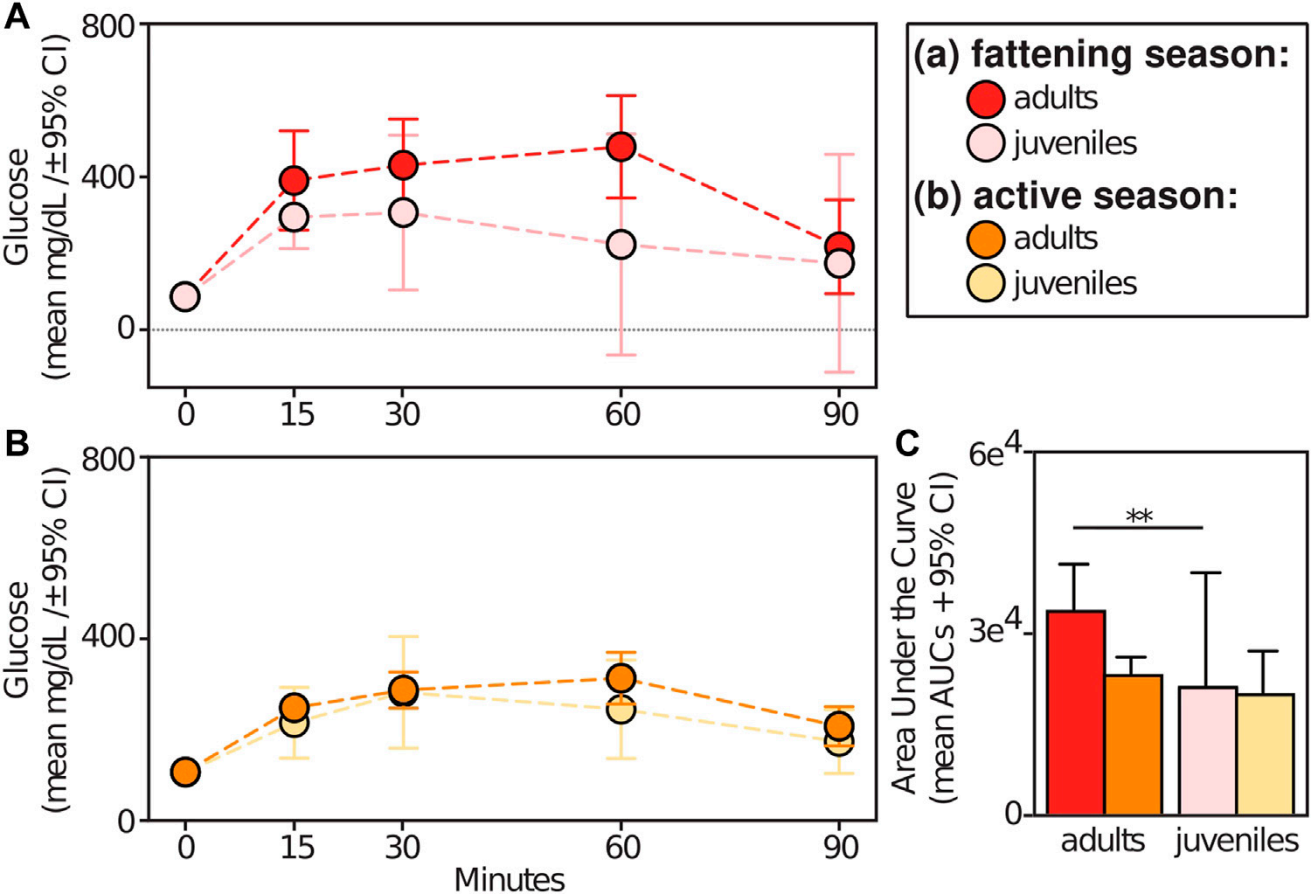
Glucose tolerance test profiles from adult vs juvenile dwarf lemurs given a dextrose challenge during the **(A)** fattening and **(B)** active seasons; **(C)** Area under the curve for adult and juveniles dwarf lemurs sampled between fattening and active seasons. **p ≤ 0.01.

Relative to animals receiving glucose tests, those receiving control tests had significantly smaller AUCs (t = −9.709; *p* < 0.001) (Figure 5A). Within experimental subjects, we found an effect of season in the response to glucose tests. Specifically, we found significantly larger AUCs during the lean *versus* active season (t = 2.313; *p* = 0.034), albeit we found no such differences between the fattening and lean season (t = 1.082; *p* = 0.293) or between the fattening and active season (t = −1.506; *p* = 0.160) (Figure 5B). When considering only the fattening (Figure 6A) and active (Figure 6B) seasons, we found that juveniles have a significantly smaller AUC compared to adults during the fattening season (t = −2.833, *p* = 0.013), but not during the active season (t = −1.022, *p* = 0.323) (Figure 6C).

## 4 Discussion

In the first study of glucose and insulin dynamics in dwarf lemurs, we find seasonal patterns that are broadly consistent with those known from other hibernating mammals. Like rodents, bears, and hedgehogs (Hoo-Paris et al., 1978; Melnyk et al., 1983; Moreau-Hamsany et al., 1988; Tokuyama et al., 1991; Rigano et al., 2017), dwarf lemurs can show signatures of lean-season insulin resistance, including elevated fasting insulin and HOMA-IR values, lower fasting glucose, and reduced glucose tolerance compared the fattening and/or active seasons. Despite variation between individuals, sexes, and age classes, dwarf lemurs during the fattening season showed patterns that bore greater resemblance to the dynamics of bears, hedgehogs, and mouse lemurs (Laurila and Suomalainen, 1974; Rigano et al., 2017; Terrien et al., 2018) compared to hibernating rodents (Tokuyama et al., 1991; Wu et al., 2013) during the same time of year; namely, they had relatively low values of glucose, insulin, and HOMA-IR. By contrast in the active season after hibernation, the lemurs showed greater fasting glucose, insulin, and HOMA-IR, as well as the greatest glucose tolerance of any timepoint. These seasonal differences highlight the processes at play during periods of weight gain *versus* loss, as animals balance carbohydrate and lipid-based metabolism. Notably during fattening, insulin likely responded to the glucose from food carbohydrates needed to deposit lipids. During the active season, insulin was likely also regulated by the adipose-tissue insensitivity needed for lipolysis to deplete any residual fat reserves.

During the lean season, two of our study conditions mimic the natural settings for wild dwarf lemurs in Madagascar- stable ambient temperatures or daily fluctuating temperatures, both without food (Dausmann et al., 2004). Under the former, dwarf lemurs cycle between torpor and arousal and showed fasting glucose and insulin dynamics that were most consistent with those from temperate hibernators: Glucose values were lowest during torpor and increased during arousals and fasting insulin was variable across individuals, likely because they were at different stages of the arousal process when sampled. Thermoconforming lemurs likewise had low fasting glucose, but curiously also had consistently low fasting insulin. This insulin result raises interesting questions about lipid-based metabolism in hibernators under fluctuating temperatures that allow them to passively cool and warm daily rather than rely on expensive and sporadic arousals from cold temperatures. Interestingly, HOMA-IR values did not differ significantly between these two conditions and remained under the standard threshold of 2.0 for insulin sensitivity, albeit the average value of 1.95 for animals under cold conditions approached this threshold, while the average value of 1.17 for animals under fluctuating conditions did not.

The other two lean-season conditions in our study are unlike anything wild dwarf lemurs would experience and involve the provisioning of daily food under stable temperature conditions. As would be expected, fasting glucose values were greater in both study groups with food compared to those without food; and fed animals under warm *versus* cold conditions had the greatest values of all. Indeed, we found HOMA-IR values well above the threshold for insulin resistance, as well as the greatest glucose intolerance, in animals kept under warm conditions with food during the lean season, highlighting that those with the greatest insulin insensitivity were also those under the least “natural” conditions. Although we were unable to perform glucose tolerance tests in food-restricted animals under different regimes during the lean season, we would expect that any dwarf lemur, like mouse lemurs (Terrien et al., 2018), undergoing weight loss and lipid metabolism to experience some insulin insensitivity, albeit perhaps to varying degrees.

Although our results from the lean season paint a relatively clear picture of glucose and insulin dynamics, those from the other two seasons are somewhat noisier. Notably, both the fattening and active seasons are times of transition: At any single timepoint, animals will show individual differences in food intake, degree of fattening, and activity levels. Such variation could explain the difference in glucose tolerance we found between adults and juveniles during the fattening season: Juveniles that were still prioritizing growth while also depositing fat were likely more sensitive to insulin to regulate glucose uptake (Boswell et al., 1994). In contrast, adults nearing their full capacity for fat stores were perhaps already showing signs of the insulin insensitivity that would characterize the forthcoming lean season. Future studies could beneficially culture adipose tissues from dwarf lemurs collected across seasons and age classes, and grown under different temperature and endocrine environments (e.g., Moreau-Hamsany et al., 1988), to more directly measure insulin resistance and the finely tuned relationships between glucose, lipids, and insulin. Moreover, monitoring of insulin levels after controlled injections could aid in the discussion of insulin resistance in these lemurs, although techniques will need to be refined to allow for sequential blood draws in these small-bodied and critically endangered animals.

While acknowledging the constraints of our study system and limits to acceptable experimentation, our results demonstrate the promise of dwarf lemurs for investigating timely questions in hibernation metabolism, including elucidating the proximate mechanisms that regulate feast-fast cycles. Like all hibernators, dwarf lemurs have circannual rhythms that establish seasonal and consistent cycles of insulin sensitivity and insensitivity. Unlike temperate hibernators, however, dwarf lemurs show metabolic flexibility during the lean season that is intrinsically linked to environmental heterogeneity (Dausmann et al., 2004; Blanco et al., 2018). This flexibility includes capacity to depress metabolism, and even hibernate, under relatively warm and fluctuating conditions. Understanding the metabolic processes at play in thermoconforming dwarf lemurs, where significant lipolysis occurs without animals having to undergo costly arousals, may yield new insights into the role of insulin and other hormones in mediating lipid-based metabolism more broadly. As primate models, hibernating dwarf lemurs add another layer to the conversation about how insulin resistance can be reversed, with clear implications for human health.

## Data Availability

The raw data supporting the conclusions of this article will be made available by the authors, without undue reservation.
